# Workplace violence against medical staff of Chinese children's hospitals: A cross-sectional study

**DOI:** 10.1371/journal.pone.0179373

**Published:** 2017-06-13

**Authors:** Zhe Li, Chun-mei Yan, Lei Shi, Hui-tong Mu, Xin Li, An-qi Li, Cheng-song Zhao, Tao Sun, Lei Gao, Li-hua Fan, Yi Mu

**Affiliations:** 1 Department of Health Management, School of Public Health, Harbin Medical University, Harbin, Heilongjiang, People’s Republic of China; 2 Department of Customer Service, Beijing Children’s Hospital, Capital Medical University, Beijing, People’s Republic of China; 3 Hospital Administration Office, Beijing Children’s Hospital, Capital Medical University, Beijing, People’s Republic of China; Old Dominion University, UNITED STATES

## Abstract

**Background:**

In China, medical staff of children’s hospitals are commonly exposed to violence. However, few studies on medical violence are conducted in the settings of children’s hospitals. The aim of this study is to assess the incidence, magnitude, consequences, and potential risk factors of workplace violence (WPV) against medical staff of children’s hospitals.

**Methods:**

A retrospective cross-sectional design was used. A self-administered questionnaire was utilized to collect data on 12 children’s hospitals. The questionnaires were distributed to a stratified proportional random sample of 2,400 medical staff; 1,932 valid questionnaires were collected. A chi-square test and multiple logistic regression analysis were conducted.

**Results:**

A total of 68.6% of respondents had experienced at least one WPV incident involving non-physical and/or physical violence in the past year. The perpetrators were mainly family members of patients (94.9%). Most of the WPV occurred during the day shift (70.7%) and in wards (41.8%). Males were 1.979 times (95% CI, 1.378 to 2.841) more likely than females to experience physical violence. Emergency departments were more exposed to physical violence than other departments. Oncology was 2.733 times (95% CI, 1.126 to 6.633) more exposed to non-physical violence than the emergency department. As a result of WPV, victims felt aggrieved and angry, work enthusiasm declined, and work efficiency was reduced. However, only 5.6% of the victims received psychological counseling.

**Conclusion:**

Medical staff are at high risk of violence in China’s children’s hospitals. Hospital administrators and related departments should pay attention to the consequences of these incidents. There is a need for preventive measures to protect medical staff and provide a safer workplace environment. Our results can provide reference information for intervention strategies and safety measures.

## Introduction

In recent years, workplace violence (WPV) in the health sector has caused widespread concern around the world. It has become a global public health problem that seriously endangers the health of medical workers and undermines the medical service environment [1, 2].

WPV is defined as any intended or actual use of power (either physical or psychological) to injure, threaten, or assault a person in a work context [2]. It is categorized into physical and non-physical (psychological) violence. All types of violence entail different degrees of damage. Physical violence involves the use of physical force against someone or the use of an object to attack someone. It includes punching, kicking, slapping, pushing, biting, pinching, wounding using sharp objects, sexual assault, and rape. It can lead to physical injury, dysfunction, permanent disability, or to no injuries at all [3]. Non-physical violence can include abuse, insults, threats, or sexual harassment [4]; it does not cause physical injury, but can cause psychological harm, such as depression, anxiety, low job satisfaction, and low work efficiency [5, 6]. Oztunc [7] found that Turkish nurses perceived verbal violence as a cause of emotional fatigue and reduced creativity, and stated that it affected their ability to provide care. Kwok [8] found that WPV could have a negative impact on job satisfaction and job performance among nurses in Hong Kong [8].

Numerous studies have found high levels of WPV among health workers [9, 10, 11, 12, 13, 14]. In a large mental health center in Israel, 88.1% of nurses reported verbal violence from patients, and 58.4% had experienced physical violence in the past year [15]. In Palestinian public hospitals, 80.4% of nurses reported exposure to violence in the previous year; 20.8% was physical and 59.6% non-physical [16]. In Australia, a retrospective study showed that verbal abuse (71%) was more common than physical abuse (29%) [17]. In China, most existing research focuses on tertiary hospitals and county-level and emergency departments of general hospitals; research shows that emergency departments have a high incidence of WPV [18, 19, 20, 21]. Research also shows that 7.8% of nurses report physically violent experiences and 71.9% report non-physically violent experiences in the preceding year (a total of 588 nurses) [22]. Among the few studies conducted in a community setting, a survey of 1,404 health care workers from Community Health Centers in Guangzhou and Shenzhen showed that 51.64% had experienced WPV [23].

In China, since the “Ordinary College Undergraduate Professional Directive,” which ended the pediatrics profession in 1998, most medical colleges stopped taking enrollments for pediatrics, resulting in shortages in the pediatric talent base, a total shortage of pediatric medical resources, a distribution imbalance, and an insufficient supply of medical personnel[24]. Furthermore, the results of the sixth national census show that there are more than 220 million children in China aged 14 and under, accounting for 16.6% of the total population. After the formal implementation of the two-child policy, there are now an estimated 3 million [25] newborns each year. However, data from the *2015 Chinese Health Statistics Yearbook* show that for every thousand children there are, on average, only 0.43 pediatricians. This means there is 1 pediatrician for every 2,300 child patients [26]. Medical staff of children’s hospitals and general pediatric hospitals thus face a high workload; this can aggravate the imbalance between supply and demand and the tension in the doctor-patient relationship. In addition, when children have complicated medical conditions, or when their condition is poor and the illness cannot be diagnosed, some parents mistrust and denounce medical staff during the treatment process, with some even taking extreme action [27]. In short, China’s particular national condition, as well as the specific situation for children, make the doctor-patient relationship unusually intense and contribute to WPV in children’s hospitals. In China, a study was conducted on WPV experienced by triage nurses at Shenzhen City Children’s Hospital. Thirty-eight nurses suffered 136 violent incidents, including 97 incidents of abuse, 30 incidents of threats, and 9 incidents of physical assault (January 2008–December 2010) [28].

In recent years, WPV in hospitals has become more frequent and severe. In the face of this violence, the normal medical order has been seriously damaged. In 2012, the Chinese Ministry of Health and the Ministry of Public Security jointly issued a notice “on the maintenance of medical institutions’ order”; in July 2014, the Supreme People's Court of China, the Supreme People’s Procuratorate, the Ministry of Public Security, the Ministry of Justice, National Health, and the Family Planning Commission of the PRC jointly formulated “Regulations on the punishment of illegal crime concerning medical services to maintain health institutions’ normal order” (hereinafter referred to as the “opinions”) to carry out one-year of maintenance of the medical order to combat criminal medical special actions. Subsequently, in September 2014, several departments issued notices on creating “safer hospital” activities, and further cracked down on illegal medical proceedings to maintain the normal medical order. To a certain extent, the implementation of these policies has curbed the increasing trend of medical offenses, and the number of criminal cases in medical institutions is declining. However, in the past two years, the phenomenon of WPV among medical staff in children's hospitals has become more common. According to incomplete statistics from January 2015 to July 2016, the media reported seven cases of serious injuries in pediatric and children’s hospitals, and the number of violent incidents was second only to that of the emergency departments of general hospitals. Thus, it can be seen that the implementation of policies such as the “opinions” has not fundamentally curbed violent injuries. Further, although WPV in children’s hospitals is grim indeed, there have been few studies on the phenomenon.

## Materials and methods

### Study design and population

A retrospective cross-sectional design was used. Data were collected between March and May 2016. The survey was conducted in the Beijing Children’s Hospital Group, which includes 12 children’s hospitals. Among them, Children's Hospitals 9–12 in Table 1 are also local maternal and child care service centers, including children’s departments, and women’s health care departments, but we selected only the medical staff of the children’s departments as the object of our investigation.

**Table 1:** 12 Children's hospital.

Number	Hospital name	Location	Characteristics
**1**	Beijing Children’s Hospital*	Beijing	a pure children’s hospital
**2**	Dalian Children’s Hospital*	Dalian city, Liaoning province	a pure children’s hospital
**3**	Liaocheng Children’s Hospital*	Liaocheng city, Shandong Province	a pure children’s hospital
**4**	Zhengzhou Children’s Hospital*	Zhengzhou city, Henan Province	a pure children’s hospital
**5**	Anhui Province Children’s Hospital*	Hefei city, Anhui Province	a pure children’s hospital
**6**	Hangzhou Children’s Hospital*	Hangzhou city, Zhejiang Province	a pure children’s hospital
**7**	Xi’an Children’s Hospital*	Xi’an city, Shanxi Province	a pure children’s hospital
**8**	Jiangxi Province Children’s Hospital*	Nanchang city, Jiangxi Province	a pure children’s hospital
**9**	Shanxi Province Children’s Hospital*	Taiyuan city, Shanxi Province	the one combing with women’s care
**10**	Wuhan Children’s Hospital*	Wuhan city, Hubei Province	the one combing with women’s care
**11**	Guiyang Children’s Hospital*	Guiyang city, Guizhou Province	the one combing with women’s care
**12**	Liuzhou Children’s Hospital*	Liuzhou city, Guangxi Province	the one combing with women’s care

* These hospitals are tertiary hospitals and are located in eastern, central, and western china.

In Beijing Children’s Hospital Group, the number of medical staff is about 11,600; we extracted a total of 2,400 samples, with these staff accounting for 20% of the total medical staff. On average, 200 medical staff from each hospital were extracted (in the actual sampling process, the sample size of each hospital was slightly different, but all were roughly 200 people). In Beijing Children’s Hospital Group, the majority of children's hospital departments and the number of formal staff are similar. Therefore, a proportionate stratified random sample was taken according to the number of Beijing Children's Hospital departments; the number of personnel taken from the emergency department was 25, while 50 staff in internal medicine were selected, as well as 50 in surgery; 5 in oncology; 20 in neonatal; 35 in ear, nose, and throat (ENT); and 15 in inspection (12 hospitals were sampled in accordance with the above ratio, in which the individual departments in the sample size were slightly different, but the basic values were as above). The study population consisted of licensed physicians, nurses, and medical technicians working on a full-time basis, but did not include trainees. A total sample of 1,320 nurses, 960 doctors, and 120 technicians was achieved.

### Data collection

The survey was conducted in March–May 2016. Consultation concerning access took place with the administrator of the Beijing Children’s Hospital Group and each study hospital. After providing consent, respondents completed and returned an anonymous questionnaire (names/identifiers not requested) to a box in the office of the department director. A total of 2,400 questionnaires were distributed; 2,084 were returned, and 1,932 valid questionnaires were selected (total response rate: 86.8%; total rate of valid questionnaires: 92.7%).

### Questionnaire

In this study, work-related violence was defined as any activity associated with the job or any event that occurred in the work environment that involved the intentional use of physical force or emotional abuse against an employee and resulted in physical or emotional injury and consequences [29, 30]. We divided WPV into non-physical and physical violence.

The study instrument was based on questionnaires used in two earlier studies [29, 30], as well as the international questionnaire “Workplace Violence in the Health Sector” developed by the International Labor Organization, the World Health Organization, the International Council of Nurses, and Public Services International. The questionnaire was modified to fit the research contents and objectives, and was translated into Chinese. To enhance its validity, it was reviewed by 15 experts, including a chief physician, a chief nurse, a hospital administrator, health administrative department experts, epidemiologists, health management experts, and public health specialists. Finally, the reliability of the questionnaire was confirmed using a two-week test-retest (r = 0.92) taken by 50 medical staff, who were then excluded from the study. Data were collected using the final questionnaire, which comprised the following sections:

1. Sociodemographic data of the participants (including gender, age, education level, marital status, professional title, employment form, monthly income, profession, department, work experience, treatment time, and contact time with the patient).
2. Experience of non-physical or physical violence within the past 12 months. Non-physical violence includes abuse, threats, and sexual harassment. Physical violence involves the use of physical force against someone or the use of an object to attack someone. It includes punching, kicking, slapping, pushing, biting, pinching, or wounding using sharp objects (multiple responses were possible).
3. If WPV in a hospital had been experienced in the past year, respondents were asked to describe one of the most memorable events, including the place and time of the event (places include outpatient departments, wards, doctors’ offices, nurses stations, therapeutic rooms, and elsewhere. The time includes the day shift, the night shift, and after work). Victim’s coping style and psychosomatic effects, and victim’s views on the causes of hospital WPV were requested, as were the characteristics of perpetrators (identity, age, and gender). The characteristics of the perpetrators and the victim’s coping style, effects, and victim’s views were all multiple-choice responses. In this section, we asked the respondents to mention only one of the most impressive encounters, so the time and place of the event were a single choice. In China, it is common for many family members to accompany children on hospital visits; therefore, the characteristics of the perpetrators (gender, identity, and age) were included as multiple-choice options.

### Data analyses

EpiData version 3.1 was used for double data entry to ensure data accuracy. Descriptive statistics were completed relating to the respondents’ demographic characteristics, years of work, and so on. Pearson’s chi-square test was used for univariate analysis to test whether exposure to violence occurred according to the demographic characteristics of the respondents, years of work, and so on. Based on the results of this test, the significant factors were used as covariates, exposure to violence (as the dependent variable) was used in the logistic regression model, and a multivariate backward stepwise regression analysis was used to calculate the odds ratio (OR) of WPV experiences and the 95% confidence intervals (CIs) for the ORs (WPV including non-physical violence and physical violence, and the chi-square test and logistic regression analysis were used, respectively). Data were analyzed using IBM SPSS Statistics 21.0, and *p*< 0.05 was considered statistically significant.

### Ethical approval

The study was reviewed and approved by the Medical Ethics Committee of Harbin Medical University College of Public Health. Before the survey, approval was also obtained from each study hospital. All participants participated voluntarily and anonymously, and provided their written informed consent.

## Results

### Background characteristics of the participants (n = 1932)

The study respondents’ characteristics are provided in Table 2. The respondents were predominantly female (79.1%), under 41 years old (83.6%), married (65.6%), and held a bachelor’s degree or higher (82.1%). Respondents were almost all either physicians (45.7%) or nurses (50.6%). Most had 1 to 10 years of experience in the profession (63.5%) and were formal employees (74.1%). They came from various departments. Approximately two-thirds of respondents spent up to eight hours or more (62.6%) of diagnosis time in the hospital each day, and the duration of direct contact with patients was six to eight hours (74.3%) (Table 2).

**Table 2:** Background characteristics of the participants (n = 1932).

Characteristic	N	Percent
**Gender**		
Male	404	20.9
Female	1528	79.1
**Age (years)**		
≤30	913	47.3
31–40	702	36.3
41–50	248	12.8
51–60	64	3.3
>60	5	0.3
**Level of education**		
<Bachelor	347	18.0
Bachelor	1072	55.5
≥Master	513	26.6
**Marital status**		
Married	1267	65.6
Single	646	33.4
Other	19	1.0
**Professional title**		
Junior	1190	61.6
Intermediate	510	26.4
Senior	232	12.0
**Employment form**		
Formal employee	1431	74.1
Temporary employee	501	25.9
**Monthly income(RMB)**		
<3000	385	19.9
3000–5000	1051	54.4
5000–10000	424	21.9
>10000	72	3.7
**Profession**		
Physician	882	45.7
Nurse	977	50.6
Medical Technician	73	3.8
**Department**		
Emergency	240	12.4
Inter Medical	468	24.2
Surgical	418	21.6
Oncology	52	2.7
Neonate	218	11.3
ENT*	376	19.5
Medical Imaging	160	8.3
**Years in the profession (years)**		
<1	205	10.6
1–4	564	29.2
5–10	663	34.3
11–20	265	13.7
>20	235	12.2
**Treatment Time(hours)**		
0–2	14	0.7
2–4	9	0.5
4–6	56	2.9
6–8	644	33.3
>8	1209	62.6
**The contact time with the patient (hours)**		
0–2	87	4.5
2–4	138	7.1
4–6	272	14.1
6–8	1435	74.3

*ENT: ears, nose, and throat

### Incidence of WPV

Of the 1,932 participants who completed the survey, 1,326 (68.6%) had experienced at least one WPV incident of non-physical and/or physical violence in the past year. Of the various types of violence, 1,316 (68.1%) respondents reported exposure to non-physical violence once or more and 206 (10.7%) reported exposure to physical violence once or more (Table 3).

**Table 3:** Incidence of exposure to workplace violence(n = 1932).

Exposure To Violence	Physical violence		Non-physical violence								Overall incidence	
			Verbal		Threats		Sexual		Total Non-physical			
	N	%	N	%	N	%	N	%	N	%	N	%
Yes	206	10.7	1276	66.0	728	37.7	15	0.8	1316	68.1	1326	68.6
No	1726	89.3	656	34.0	1204	62.3	1917	99.2	616	31.9	606	31.4

Table 4 shows the descriptive association between respondents’ characteristics and exposure to non-physical or physical violence in the past 12 months. The results indicated that more males than females had been exposed to the two types of violence. Married respondents reported more non-physical violence (72.9%, *p* = 0.000) and physical violence (12.4%, *p* = 0.001). In total, 75.1% of physicians reported non-physical violence (*p* = 0.000); however, there was no statistical significance in relation to reported physical violence by profession (*p* = 0.223). Furthermore, the highest risk of non-physical violence was in oncology (86.5%), followed by ENT (77.1%) and internal medicine (71.6%), whereas the highest incidence of physical violence (18.3%) was in emergency departments, followed by oncology (15.4%) and medical imaging (12.5%). More specifically, respondents who had more than eight hours of treatment time suffered more non-physical violence (*p* = 0.000).

**Table 4:** Characteristics of exposures to physical and non-physical violence in the last 12 months (n = 1932).

	N	Non-physical violence*				Physical violence			
		n	%	χ^2^	P-value	n	%	χ	P-value
**Gender**									
Male	404	303	75.0	11.146	0.001	64	15.8	14.384	0.000
Female	1528	1013	66.3			142	9.3		
**Age (**years**)**									
≤30	913	553	60.6	46.019	0.000	76	8.3	11.962	0.008
31–40	702	527	75.1			96	13.7		
41–50	248	182	73.4			27	10.9		
≥51	69	54	78.3			7	10.1		
**Education**									
<Bachelor	347	194	55.9	44.236	0.000	28	8.1	6.252	0.044
Bachelor	1072	725	67.6			110	10.3		
≥Master	513	397	77.4			68	13.3		
**Marital status**									
Married	1267	924	72.9	39.249	0.000	157	12.4	11.551	0.001
Single or other	665	392	58.9			49	7.4		
**Professional title**									
Primary	1190	757	63.6	28.940	0.000	111	9.3	6.511	0.039
Middle	510	385	75.5			62	12.2		
Senior	232	174	75.0			33	14.2		
**Employment form**									
Formal employee	1431	1025	71.6	31.345	0.000	161	11.3	2.005	0.157
Temporary employee	501	291	58.1			45	9.0		
**Monthly income(RMB)**									
<3000	385	227	59.0	26.581	0.000	40	10.4	8.318	0.040
3000–5000	1051	716	68.1			99	9.4		
5000–10000	424	317	74.8			61	14.4		
>10000	72	56	77.8			6	8.3		
**Profession**									
Physician	882	662	75.1	36.843	0.000	103	11.7	3.003	0.223
Nurse	977	605	61.9			93	9.5		
Medical Technician	73	49	67.1			10	13.7		
**Department**									
Emergency	240	167	69.6	65.859	0.000	44	18.3	26.770	0.000
Internal medicine	468	335	71.6			53	11.3		
Surgery	418	256	61.2			31	7.4		
Oncology	52	45	86.5			8	15.4		
Neonate	218	110	50.5			13	6.0		
ENT	376	290	77.1			37	9.8		
Medical Imaging	160	113	70.6			20	12.5		
**Work experience(years)**									
<1	205	82	40.0	96.017	0.000	6	2.9	22.840	0.000
1–4	564	375	66.5			53	9.4		
5–10	663	484	73.0			90	13.6		
11–20	265	207	78.1			36	13.6		
>20	235	168	71.5			21	8.9		
**Treatment Time(hours)**									
0–6	79	33	41.8	55.837	0.000	6	7.6	1.322	0.516
6–8	644	394	61.2			65	10.1		
>8	1209	889	73.5			135	11.2		
**The contact time with the patient(hours)**									
0–2	87	39	44.8	23.798	0.000	3	3.4	8.451	0.038
2–4	138	96	69.6			10	7.2		
4–6	272	181	66.5			36	13.2		
6–8	1435	1000	69.7			157	10.9		

* Nonphysical violence includes threats, verbal abuse and sexual harassment.

χ2: Pearson Chi-Square Test

### Factors associated with WPV

The multiple logistic regression analysis results for factors associated with experiencing WPV are presented in Table 5. An increased likelihood of non-physical violence was predicted among physicians who held a master’s degree and provided more than eight hours of treatment time every day. In addition, those in an oncology department were 2.733 times (95% CI, 1.126–6.633) more exposed to non-physical violence than were those in an emergency department. Males working in an emergency department were more likely to suffer from physical violence than from non-physical violence. Similarly, those with 11–20 years of work experience were at the highest risk of experiencing both non-physical and physical violence.

**Table 5:** Multiple logistic regression of non-physical violence (n = 1315) and physical violence (n = 206).

	Non-physical violence			Physicalviolence		
	OR	95% CI	P-value	OR	95% CI	P-value
**Gender**						
Female				1.0	Reference	
Male				1.979	1.378–2.841	0.000
**Education**						
<Bachelor	1.0	Reference		1.0	Reference	
Bachelor	1.203	0.908–1.592	0.198	1.020	0.647–1.607	0.932
≥Master	1.734	1.149–2.617	0.009	1.574	0.951–2.605	0.078
**Monthly income (RMB)**						
<3000				1.0	Reference	
3000–5000				0.621	0.409–0.941	0.025
5000–1000				0.819	0.506–1.327	0.417
>10000				0.363	0.137–0.960	0.041
**Profession**						
Physician	1.0	Reference				
Nurse	0.664	0.497–0.888	0.006			
Medical Technician	0.729	0.396–1.342	0.309			
**Department**						
Emergency	1.0	Reference		1.0	Reference	
Internalmedicine	1.054	0.735–1.511	0.775	0.553	0.353–0.866	0.010
Surgery	0.650	0.454–0.932	0.019	0.284	0.169–0.478	0.000
Oncology	2.733	1.126–6.633	0.026	0.770	0.332–1.785	0.543
Neonate	0.392	0.260–0.592	0.000	0.287	0.148–0.557	0.000
ENT	1.408	0.953–2.078	0.086	0.438	0.269–0.713	0.001
Medical Imaging	1.016	0.611–1.689	0.952	0.560	0.306–1.028	0.061
**Work Experience(years)**						
<1	1.0	Reference		1.0	Reference	
1–4	3.116	2.193–4.429	0.000	3.703	1.534–8.938	0.004
5–10	4.443	3.112–6.342	0.000	6.304	2.610–15.229	0.000
11–20	5.398	3.512–8.298	0.000	6.468	2.538–16.485	0.000
>20	4.108	2.676–6.306	0.000	4.377	1.636–11.706	0.003
**Treatment Time(hours)**						
0–6	1.0	Reference				
6–8	1.603	0.934–2.751	0.087			
>8	2.633	1.554–4.459	0.000			
**The contact time with the patient(hours)**						
0–2	1.0	Reference		1.0	Reference	
2–4	1.995	1.081–3.681	0.027	1.664	0.433–6.388	0.458
4–6	2.025	1.173–3.496	0.011	3.232	0.943–11.077	0.062
6–8	2.216	1.360–3.611	0.001	2.857	0.867–9.412	0.084

OR: Odds ratios, CI: Confidence interval, Reference: reference category in the logistic regression model

### Characteristics of perpetrators, and the time and place of WPV

Table 6 shows the characteristics of perpetrators and the time and place associated with WPV. The respondents described perpetrators of WPV as mainly family members (94.9%). Only 72 (5.4%) of the perpetrators were the patients themselves. Most of the perpetrators were males (83.8%) aged 31–40 years (63.7%).

**Table 6:** Characteristics of perpetrators, time and place associated with workplace violence.

Item	Variable	Frequency	Percent
**perpetrators**			
	Patient	72	5.4
	Family members	1258	94.9
	Visitors	268	20.2
	Other	16	1.2
**Perpetrators of gender**			
	Male	1110	83.8
	Female	776	58.6
**Perpetrators of age**			
	≤20years	37	2.8
	21–30years	533	40.2
	31–40years	844	63.7
	41–50years	308	23.2
	51–60years	231	17.4
	>60years	67	5.1
**Location of the incident**			
	Outpatients Department	373	28.1
	Ward	554	41.8
	Doctor's Office	156	11.8
	Nursing station/office	158	11.9
	Treatment room	51	3.8
	Other	34	2.6
**Time of occurrence**			
	Day shift	937	70.7
	Night shift	362	27.3
	After work	27	2.0
**When the event occurs**			
	Alone	418	31.5
	There are other colleagues present	908	68.5

The characteristics of the perpetrators were multiple-choice(identity, gender and age). The time and place of occurrence is a single-choice.

Most of the violence occurred during the day shift (70.7%). Wards (41.8%) and outpatient departments (28.1%) were the most common locations of incidents.

### Victim’s coping style and psychosomatic effects, and victim’s views on the causes of hospital WPV

Table 7 shows the victim’s coping style, any psychosomatic effects, and victim’s views on the causes of hospital WPV. The majority of the victims adopted a coping style involving patience and understanding (56%), tolerance and avoidance (55.5%), asking a manager or security for help (45.7%), or asking a colleague for help (27.8%). The most frequent consequences of violent assault were aggrievement (62.4%), anger (59.8%), a decline in work enthusiasm (56.9%), a decline in work efficiency (43.8%), and an inability to concentrate (40.3%). After the violent incident, only 2.7% of the victims received conventional therapy or psychological counseling (5.6%); most of the victims received no treatment at all.

**Table 7:** Victim's coping style and psychosomatic effect, victims' views on the causes of hospital workplace violence.

Item	Frequency	Percent
**Coping**		
Tolerance and avoidance	736	55.5
Patience and understanding	743	56.0
Give tit for tat(such as sparring,insults)	34	2.6
Afterthefirstreasonablecounterattack	258	19.5
Ask a colleague for help	369	27.8
Ask a managers or security for help	606	45.7
Ask a other patients or family member for help	39	2.9
Call the police	278	21.0
Other	24	1.8
The influence of psychology andbehavior		
No effect	95	7.2
Unable to concentrate	535	40.3
Aggrievement	828	62.4
Angry	793	59.8
Anxious	398	30.0
Depressed	322	24.3
Insomnia	280	21.1
Work enthusiasm decline	755	56.9
Work efficiency decline	581	43.8
Hate and fear of patients and their families	298	22.5
Interpersonal communication barriers	112	8.4
Have turnover intention	334	25.2
Havesuicidalthoughts	34	2.6
Scene repetition	160	12.1
Other	11	0.8
Because of this event to see a doctor		
Psychological counseling	74	5.6
Routine treatment	36	2.7
No	1216	91.7
The causes of perpetrators initiated violence		
Patient disability or death	95	7.2
Patients with dysfunction or complications	100	7.5
Mental disorders	66	5.0
Drinking and drug abuse	166	12.5
Low education level	689	52.0
Long waiting time for treatment	498	37.6
Unreasonable demands are rejected	597	45.0
Not satisfied with treatment effect	582	43.9
Not satisfied with the service attitude of medical staff	338	25.5
The hospital service process is cumbersome	434	32.7
Medical expenses are expensive	403	30.4
Want to seek economic compensation	248	18.7
Other	76	5.7

Among the 1,326 victims, 52% of respondents blamed the incidents on perpetrators’ low levels of education; 45% thought it was because unreasonable demands from the patient’s family were rejected; 43.9% thought that those on the patient’s side of the family were not satisfied with the results of the treatment; 37.6% thought the incident was because of the long wait for treatment; and 32.7% thought it was because the hospital service process was cumbersome.

## Discussion

### Incidence and magnitude of workplace violence

Through our investigation of 12 children’s hospitals in China, we found that 68.6% of medical staff had suffered at least one WPV incident over the past year, higher than in previous studies of pediatric care [31]; this indicates that the problem is growing. The reasons for this are as follows: (1) since 1998, the abolition of the pediatric profession and changes to the pediatric training mechanism resulted in a shortage of talent and an imbalance in supply and demand [24]. (2) In general hospitals, pediatric medical resources have been shrinking and pediatric outpatient clinics have even been eliminated, resulting in a sharp increase in the number of outpatient clinics in children’s hospitals, overworked medical staff, poor service attitudes, crowded environments, and prolonged waiting times; these characteristics lead to pediatric patients’ families’ dissatisfaction and conflicts with medical staff [32]. (3) At present, most of the child patients are their family’s only child. Thus, when the patient’s condition is difficult to diagnose and/or treat, the treatment process is likely to induce stress in the patient’s family members, who find themselves in an unexpected situation [33]. These factors may lead to China’s children’s hospitals’ medical staff suffering violence more frequently.

Our results showed that 68.1% of medical staff had experienced non-physical violence, which is consistent with research results from the past (between 20–80%) [22, 34, 35, 36, 37]. We found that males were more likely to suffer WPV than females, which is consistent with some previous studies [18, 38, 39]. We also found that physicians were more vulnerable to non-physical violence than nurses, which is consistent with some previous studies [18, 37, 40], though others provided evidence of the opposite [38, 41]. The reason is that the ratio of children to pediatricians in China is 2,300 to 1 [24, 26], so obviously there are not enough pediatricians; each doctor initially examines children for an average of just 5 minutes [42]. Moreover, treatment time is too short, and pediatric patients’ families do not have sufficient time to communicate with doctors, causing the family to complain to the doctor or even engage in verbal aggression. Our study found that the highest risk of non-physical violence was in the oncology departments, and the highest risk of physical violence was in the emergency departments. Most past studies have shown that there is a high risk of violent incidents in emergency departments, psychiatric departments, and intensive care units [22, 37, 39, 43]. This may be attributed to the following two factors. First, research on empathy, fatigue, and occupational burnout shows that medical staff in oncology departments are especially susceptible to these disorders, given their unique work environment and service objective [44, 45]. Their levels of physical and mental health, work quality, and service attitude decrease and they often experience negative emotions [46, 47]. Second, tumors have a low cure rate, a high recurrence rate, high mortality, and high treatment costs. Thus, pediatric patients’ families very easily grow discontented with the results of treatment.

### Factors associated with workplace violence

Certain characteristics have been found to increase the risk of workers becoming targets of WPV in a health care setting, including the workers’ gender, age, years of experience, marital status, department, and previous WPV training [16, 37, 38, 48]. Our logistic regression results showed that education level, profession, department, work experience, treatment time, and direct contact with patients affected the incidence of non-physical violence. Moreover, gender, education level, monthly income, department, work experience, and direct contact with patients affected the occurrence of physical violence. These differences may be partially due to cultural factors, methodological differences (such as the definitions of various types of violence), or sampling techniques. We found that medical staff with 10–20 years of work experience were the most vulnerable to WPV, but previous studies showed that individuals with 10 years’ experience or less were more vulnerable to WPV [16, 19, 39]. The reason for this may be that most of them, as their main line of clinical work, are responsible for treating critically ill children and performing difficult operations that carry a significant medical risk [49]. In addition to facing the pediatric patients’ families, experienced health care workers face especially difficult work with children. Children’s small blood vessels make injections and surgeries more difficult; furthermore, they are susceptible to rapid changes in condition that increase the difficulty of diagnosis and treatment. Once an error in treatment is made by medical staff, there is a gap between the high expectations of patients’ families and the realities of treatment, resulting in patients’ families misunderstanding medical staff and even initiating violent attacks. Our study found that the longer the treatment period (which entails more contact with patients), the more vulnerable hospital staff were to non-physical violence. Survey data showed that 73.5% of medical staff who had been treating patients for more than eight hours were subjected to non-physical violence; 69.7% of those who had contact with patients for more than six hours had suffered from non-physical violence. Overtime work, work overload, and a noisy work environment affected the quality of medical staff and doctor-patient communication, thus making the doctor-patient relationship tense and contributing to WPV.

### Characteristics of perpetrators and the time and place of WPV

Detailed data on perpetrators and the time and place of incidents were gathered in this study. The results were consistent with findings in the United Kingdom [50], which reported that WPV was perpetrated mainly by males aged 20–40 from the pediatric patient’s family. The reason may be that young men are more impulsive and more likely to quarrel with medical staff or even engage in physical violence. Furthermore, most of the violence occurred on the day shift in the presence of colleagues, mainly in wards and outpatient departments. Past research results show that violence mainly occurred during the day shift, and some of the emergency department studies show that violence occurred mainly at night [49]. This may be because the occurrence of violence does not depend solely on the time of day but also on the workload and intensity of the medical staff. For children’s hospital wards or clinics, all kinds of examinations, treatments, and care measures are arranged during the day. During this time, even if in-service health care staff are still very busy, it is common for one patient to be accompanied by multiple family members. Medical staff need to meet the needs not only of patients, but also of family members; even slight carelessness may make patients or their families discontent. During the night shift, an emergency department has only a small number of health care workers on duty. There is no diversion of patients from outpatient services, so the workload is larger than on the day shift, and child patients are often hospitalized in critical condition. Thus, family members may be more irritable and may violently attack the medical staff.

### Victim’s coping style, psychosomatic effects, and victim’s views on the causes of hospital WPV

Our study found that victims believed that attacks were mainly caused by the perpetrators’ low educational level; unreasonable demands from the patient’s family being rejected; and family members being dissatisfied with the effects of treatment or the long wait times, cumbersome hospital services, and medical expenses. This is consistent with previous research results [51, 52, 53]. The specialized nature of medical knowledge and the asymmetry of medical information means that family members often lack medical knowledge and an understanding of the diagnostic process. The specificity of medical services, limitations of medical technology, shortage of high-quality medical resources, and other factors lead family members to believe that the condition has not improved, which triggers conflicts between doctors and patients. In addition, in Chinese children’s hospitals, the phenomenon of “three long and one short” (long lines for registration, waiting, and payment, but a short time with the doctor) is common, which makes patients and their relatives more anxious.

We found that when medical staff encountered WPV, the main coping styles were understanding, tolerance, and avoidance, as well as recourse to colleagues, managers, or security. A total of 91.7% of victims were not treated in any way, which is consistent with previous studies [16, 54]. Fewer medical staff chose to alert others or to retaliate, and did not intend to seek psychological counseling or routine treatment, probably because most of the violence was non-physical in nature. Even so, this does not mean there was no effect on the staff members’ physical and mental fitness. It was found that the most common psychological reactions of victims were aggrievement and anger, followed by anxiety, inability to concentrate, loss of work enthusiasm and work efficiency, and even willingness to leave their position. Related studies have reported that most medical staff who have been subjected to WPV will display various levels of post-traumatic stress disorder for several weeks or even for over a year [55]. Carroll found that in the United States, verbal violence can cause nurses to resign, which affects the quality of care that patients receive [56]. Therefore, WPV not only affects the health and work quality of medical staff but also prevents teams of health care workers from being built and affects the working order of medical institutions. Thus, governments and health managers need to pay attention to and address WPV.

At present, domestic research on WPV in hospitals has gradually increased, and the medical community is becoming more and more concerned about the issue of violent injuries. The relevant departments have strengthened management efforts and introduced relevant policies to resist WPV in hospitals. The implementation of the “opinions” and “safe hospital” activities played a positive role in the fight against illegal and criminal activities involving medical staff. The Supreme People’s Court of China and the National Health and Family Planning Commission of the PRC jointly conducted a briefing on maintaining medical order in order to build harmonious doctor-patient relationships, and the number of medical disputes and criminal cases involving medical care has “doubled down”for three consecutive years (2014–2016); for example, the number of criminal cases involving medical staff fell 14.1% in 2016 [57]. However, the phenomenon of medical staff suffering abuse and threats is still widespread, and malignant injuries occur now and then. Thus, the relevant departments need to include more in-depth treatment of abuse, threats, and physical violence at the legal and institutional levels, and genuinely implement a zero-tolerance policy toward violence. Medical staff who have suffered WPV in a hospital should be given priority for psychological counseling, to ensure their physical and mental wellbeing, and to enable them to better serve patients.

## Limitations

This study had several limitations. First, data were collected retrospectively; this method depends on the ability of participants to recall events that occurred in the 12 months before the study, potentially resulting in recall bias. Second, as the study’s time and resource restrictions confined the investigation to 2,400 medical staff from 12 tertiary children’s hospitals, the results cannot fully represent the current status of WPV in Chinese children’s hospitals. Third, random stratified sampling was adopted according to the number of departments and workers, meaning that only 73 medical technicians and 52 oncology department workers were surveyed; this may have affected our results. Fourth, we asked the victims to discuss the most serious incident in terms of its time and location; the characteristics of the perpetrators; and the coping style, behavior, and psychological effects, without dividing the instances into physical and non-physical violence; thus, the results failed to compare physical and non-physical violence. Finally, this study only focused on the current situation of WPV in children’s hospitals. To follow up, we intend to discuss the inducing factors and preventive measures of this phenomenon.

## Conclusion

The results of this study showed that the medical staff of children’s hospitals in China suffer from serious WPV, which has a negative impact on their psychology and behavior. A series of laws and regulations should be instituted to combat WPV in hospitals, the government and the health sector should develop policies and strategies to prevent WPV in hospitals, and hospital managers should provide appropriate psychological support to the victims.

## Supporting information

S1 Dataset Supporting dataset. The supporting dataset includes the data underlyingour findingsin this study.(XLS)
(XLS)
